# Mycorrhizas and soil ecosystem function of co-existing woody vegetation islands at the alpine tree line

**DOI:** 10.1007/s11104-016-3047-2

**Published:** 2016-09-12

**Authors:** Lixia Wang, Burenjargal Otgonsuren, Douglas L. Godbold

**Affiliations:** 1 Institute of Forest Ecology, University of Natural Resources and Life Sciences Vienna (BOKU), Peter-Jordan-Straße 82, 1190 Vienna, Austria; 2 Department of Ecology, Mongolian University of Life Science, Zaisan, Mailbox 57, Khan-Uul district, Ulaanbaator 17024 Mongolia; 3 Department of Landscape Carbon Deposition, Global Change Research Institute, Academy of Sciences of the Czech Republic, Na Sádkách 7, 370 05 Ceské Budejovice, Czech Republic

**Keywords:** Ectoenzymes, Ectomycorrhizas, Enzyme activity, Ericoid mycorrhizas, Nitrogen-mineralization

## Abstract

**Background and aims:**

*Picea abies*, *Pinus mugo* and *Rhododendron ferrugineum* co-exist at the alpine tree line, and can have different mycorrhizal communities. The activity and diversity of mycorrhizal fungi are considered to be important factors in regulation of soil function.

**Methods:**

At a tree line site and a lower elevation site in the Austrian Alps, the community structure of ectomycorrhiza on *Picea abies* and *Pinus mugo* was determined. The activity of surface enzymes was determined on ectomycorrhizal and ericoid mycorrhizal roots. In soils, the activity of a range of enzymes, nitrogen (N) mineralization and biomass decomposition were determined.

**Results:**

The community structure of the ectomycorrhizal community of *Picea abies* and *Pinus mugo* differed strongly, but the average activity of surface enzymes of the ectomycorrhizal communities was similar. A lower root surface enzyme activity was determined on *Rhododendron ferrugineum*. Soil N-mineralization under *Rhododendron ferrugineum* was significantly lower than under *Picea abies* and *Pinus mugo*. In soil, the activity of a range of enzymes did not differ at the tree line but differed between the tree line and the lower elevation sites.

**Conclusion:**

The different ectomycorrhizal communities on *Picea abies* and *Pinus mugo* and ericoid mycorrhizas on *Rhododendron ferrugineum* support similar ecosystem functions in soil.

**Electronic supplementary material:**

The online version of this article (doi:10.1007/s11104-016-3047-2) contains supplementary material, which is available to authorized users.

## Introduction

Tree lines are among the most conspicuous transitions between vegetation types in mountain regions worldwide and can have a number of forms (Harsch and Bader 2011). One typical treeline form is the formation of tree islands of woody vegetation in a matrix of ericaceous and alpine grassland vegetation (Haselwandter 2007). In many places in the Alps the tree islands are an advancement of the tree line due to a decrease in grazing pressure (Holtmeier and Broll 2010). In the Alps, the most abundant woody species are *Picea abies* and *Larix decidua,* and above the tree line *Pinus mugo*. Above the tree line in the silicate Alps *Rhododendron ferrugineum*, *Vaccinium myrtillus*, and *Vaccinium vitis-idaea* are the dominant ericaceous plant species, and often grow in discrete patches. The tree line ecotone also forms a transition zone for mycorrhizal forms, whereas the tree species such as *Pinus mugo*, *Pinus cembra*, *Larix decidua* and *Picea abies* all form ectomycorrhizas, the ericaceous species typically form ericoid mycorrhizas (Haselwandter 1987). In the alpine-treeline ecotone, soil moisture levels are high, with decomposition and mineralization processes inhibited as a result of low soil temperatures, high rain or snowfall and low evaporation rates (Haselwandter 2007).

Ectomycorrhizal community structure is influenced by a number of factors for example host preference, host age and soil properties (Bills et al. 1986; Di Marino 2008; Johnson et al. 2005; Kernaghan et al. 2003). Although many investigations have determined ectomycorrhizal community structure in forests (Fransson et al. 2000; Gardes and Bruns 1996; Grogan et al. 2000) there are few published reports of the structure of tree line ectomycorrhizal communities. Using surveys of sporocarps, Moser (1967, 1982) found the community of ectomycorrhizal fungi at the tree line to be an amalgamation of species from the subalpine forest and the alpine zone. Kernaghan and Harper (2001) reported that species richness of ectomycorrhizal fungi decreased with increasing elevation from the sub-alpine forest, through the tree line, to alpine tundra in the Canadian Rockies. Species richness as well as functional group diversity of ectomycorrhizal fungi are thought to be important in maintaining ecosystem function (Baxter and Dighton 2001; Cairney and Meharg 1999; Leake et al. 2001).

Ericaceous plants occur in the understory of boreal and temperate forests (Clemmensen et al. 2015), but also as pure patches above the altitudinal and latitudinal tree line (Read et al. 2004). A characteristic of ericaceous plants is that they have a litter low in N and phosphorus (P), and a function attributed to ericoid mycorrhizas is the ability to mobilize N and P from these low quality litter substrates using extracellular enzymes (Cornelissen et al. 2001). However, both ericoid and ectomycorrhizal fungi have the ability to produce extracellular enzymes to breakdown a range of organic substrates such as lignin, cellulose, proteins and organic P (Courty et al. 2010; Pritsch and Garbaye 2011; Read et al. 2004). Ericoid mycorrhizal fungi have been shown in pure cultures to have high in vitro activities of oxidative and hydrolytic enzymes for N mobilization (proteases) and P mobilization (phosphatases) as well as of enzymes involved in litter decomposition (cellulases and polyphenol oxidases) (Leake and Read 1997; Read et al. 2004). Similarly, for ectomycorrhizas production of extracellular enzymes has been shown in mesocosms, but more recently also on the surface of ectomycorrhizal root tips (Courty et al. 2010; Pritsch and Garbaye 2011). For example, ectomycorrhizal fungi were shown to produce acid phosphatase in both the hyphal mantel and the attached ramifying mycelium (Dodd et al. 1987), and are able to hydrolyze a number of complex organic P compounds (Alexander and Hardy 1981; Bartlett and Lewis 1973; Dighton 1983; Ho and Zak 1979). Measurement of a range of ectoenzymes (enzyme profiling) has been used in many studies to assess the function of ectomycorrhizas and their potential to mobilize organic nutrients (Buée et al. 2007; Pritsch and Garbaye 2011). Therefore, activities of extracellular enzymes can be considered as functional traits to study functional diversity of the ectomycorrhizal community (Cullings and Courty 2009). Between different species of ectomycorrhizas considerable differences in ectoenzyme activity have been shown (Buée et al. 2007), but also considerable plasticity within a species (Pritsch and Garbaye 2011).

Analysis of soil enzyme has been used to provide understanding of processes linking microbial populations and nutrient dynamics (Schimel and Weintraub 2003; Sinsabaugh and Moorhead 1994). Soil enzyme activities are frequently utilized to evaluate the ecological integrity of soils, and as a general indicator of microbial activity (Ken and William 2002). Soil enzymes can be secreted by both microorganisms and plants. Bacteria release phosphatases and other microbial extracellular enzymes, including proteases, amylases, glucose isomerases, pectinases and lipases (Tabatabai and Dick 2002). Plants also have been considered as a source of soil enzymes (Tabatabai and Dick 2002). Similarly, saprotrophic fungi also produce extracellular enzymes that are capable of mineralizing C, N and P from soil organic matter and litter. However, in forests, saprotrophic fungi are generally confined to fresh litter and the surface of the forest floor where C is mineralized, and mycorrhizal fungi dominate in more decomposed litter and soil where N is mobilized and made available to plants (Lindahl et al. 2007). For example, in a forest soil the activity of ectomycorrhizal fungi was highest in the H and A horizon (Voříšková et al. 2014). In forest soils, fungi have been suggested to be a controlling factor in soil enzyme activity (Burke et al. 2011). Burke et al. (2011) showed that arbuscular fungal communities were positively correlated with the activities of urease and leucine aminopeptidase, enzymes involved in N cycling, and ectomycorrhizal/saprotrophic fungal communities were positively correlated with most soil enzymes, including enzymes involved in C, N and P cycling. Baldrian (2009) suggested that many of the oxidative enzymes such as peroxidase and laccase originate from the activity of saprotrophic fungi. However, the enzyme levels in soil systems also vary in relation to the soil organic matter content (Stevenson et al. 1986).

In the present study, we tested the hypothesis that the mycorrhizal community influences soil ecosystem function. To this end, we utilized islands of co-existing woody vegetation at the alpine tree line (*Picea abies*, *Pinus mugo*, *Rhododendron ferrugineum*) and a mature forest stand (*Picea abies*) at a lower elevation. At the sites, we determined the type of mycorrhiza (ericoid or ectomycorrhizas), and for the ectomycorrhizas the community structure. To estimate the function of the mycorrhizas, we determined the surface ectoenzyme activity of the ericoid mycorrhiza and all species of ectomycorrhizas. The mycorrhizal community and ectoenzyme activity were then related to measures of soil ecosystem function determined as soil enzymes activity, N-mineralization and litter decomposition.

## Materials and methods

### Site description

Study site is located in the Wasserberg area of the Stift Heiligenkreuz forest estate in the central Alps in Austria. The work was conducted at the tree line and at a lower elevation site in a closed forest. The forest area of the Wasserberg is dominated by *Picea abies* L. Karst, as the potential natural vegetation. In July 2014, at the tree line, five replicate plots were established between 1668 and 1791 m above sea level on a southeast facing slope (47°19’N, 14°43’E). Each plot contained discrete areas dominated by *Picea abies, Pinus mugo and Rhododendron ferrugineum* (Fig. S1). The *Rhododendron ferrugineum* plots also had *Vaccinium myrtillus* growing within the more open bush parts. The plots were ca. 120 m apart. The *Picea abies* trees were ca. 4 m in height, and estimated to be ca. 30 years old, and are part of a natural encroachment of the tree line. At a lower elevation (1395 m above sea level), five replicated plots were established in a closed canopy area of the *Picea abies* forest (47°18′ 559″N, 14°45′ 271″E). The minimum distance between the plots was ca. 40 m. The trees were estimated to be between 90 and 120 years old, and ca. 40 m in height, and had a mean diameter at breast height (DBH) of ca. 50 cm. Soils at the sites are developed from gneiss, and are Dystric Cambisols with an H layer of ca. 6 cm and an A layer of ca. 10 cm (Fig. S1).

### Soil sampling

On 1st July 2014 and 12th June 2015 two soil cores per sub-plot were taken to a depth of 12 cm from each of the species sub-plots of *Picea abies, Pinus mugo* and *Rhododendron ferrugineum* at tree line site and *Picea abies* site of lower elevation using a 7 cm diameter stainless steel corer. For all the species the highest fine root density was in the upper 12 cm of the soil. Soil samples were directly returned to the laboratory and stored at 4 °C until further analysis.

### Decomposition bags

On the 1st July 2014, 2 rooibos tea and 2 green tea were inserted to 5 cm soil depth using a narrow (5 cm) planting spade at each plot. The green tea consisted of 89 % green tea (Liptons Unilever), and the rooibos tea (Liptons Unilever) consisted of 93 % rooibos, both were supplemented with natural flavorings (Keuskamp et al. 2013). Size of the nylon mesh was 0.25 mm, it allows microorganisms and mesofauna to enter the bags, but excludes macro fauna (Keuskamp et al. 2013). The tea bags were removed on 14th November 2014, and dried in the oven at 80 °C until constant weight. The soil attached to the surface of tea bags was carefully removed with a brush. The difference between the initial and post incubation weights were used for calculating the mass loss.

### Soil temperature

Temperature sensor (model DS1922L-F5, precision: 0.5 °C, accuracy: ±1 °C) were set to record temperatures every 3 hours. For installation, the sensors were wrapped in plastic bags to prevent corrosion, and buried to 5 cm beneath soil surface on 11th June 2015. The data were read using a one wire viewer on 22th July 2015. Means were calculated from the 40 day readings.

### Soil analysis

The soil of the cores taken in 2014 was sieved separately to 2 mm before analysis. Soil pH was determined on field moist soil using a 1:2 soil suspension in distilled water. Soil moisture content was determined gravimetrically, by measuring the moisture loss after drying at 80 °C for 24 h.

For analysis of total C and total N, soil dried at 80 °C was finely ground in a mortar, and C and N were determined in 100–150 mg samples using automated dry combustion (LECO TruSpec CN).

### Ectomycorrhizal morphotyping

In order to assess the ectomycorrhizal community structure, fine roots were removed from each soil core taken in 2014 to give a sample with approximately 150–300 root tips per core. The samples were then washed carefully, placed into petri-dishes filled with clean tap water, and stored at 4 °C (analyzed within three weeks). All clearly definable ectomycorrhizal root tips from each sample were sorted into morphotypes based on the method described by Agerer (1997), using a ZEISS (Stemi 2000-CS) dissecting microscope which was connected with an AxioCam ERc5s camera. The final identification to genus or species level (where possible) was carried out by sequencing of DNA (see below). The total number of root tips colonized by each of the morphotype was counted under the dissecting microscope. Between 1 and 10 ectomycorrhizal root tips of each morphotype were placed into micro-centrifuge tubes. The number of root tips varied from one to ten depending on the abundance of the morphotype. The samples were then stored at −20 °C until DNA extraction.

### DNA extraction and PCR amplification

The 1.5 ml micro-centrifuge tubes containing the ectomycorrhizal root tips were placed in liquid N for 5–10 min, and the tips were ground with a sterilized glass bar. DNA from the crushed ectomycorrhizal root tips was extracted by using DNeasy Plant Mini kits (QIAGEN), and the extracted DNA was stored at −20 °C until the PCR reactions were run. For the PCR reactions, 1 μl DNA template was mixed with 12.5 μl MyTaq mix (BIOLINE), 0.5 μl ITS1F (20 μM) primer (CTTGGTCATTTAGAGGAAGTAA forward), 0.5 μl ITS4 (20 μM) primer (TCCTCCGCTTATTGATATGC reverse), and diluted with 10.5 μl distilled deionized H_2_O. For the PCR, the Thermocyler (TProfessional Basic) cycling parameters were an initial denaturation at 95 °C for 1 min, a second denaturation at 94 °C for 30 s, annealing at 50 °C for 40 s, and extension at 72 °C for 30 s, followed by a final auto-extension step at 72 °C for 4.5 min. The step from the second denaturation to extension was run for 35 cycles. To check the success of the PCR amplification, electrophoresis was carried out using 1 % regular agarose gel stained with SERVA DNA Stain G in a 1 % Tris-EDTA buffer solution. The gel was then visualized under UV light. If a clear single band was visible on the gel, the PCR products were sent for sequencing. Sequencing was done by Macrogen Inc., Seoul, Korea. Sequencing reactions were performed in a MJ Research PTC-225 Peltier Thermal Cycler using a ABI PRISM® BigDyeTM Terminator Cycle Sequencing Kits with AmpliTaq® DNA polymerase (FS enzyme) (Applied Biosystems), following the protocols supplied by the manufacturer. Single-pass sequencing was performed on each template using an ITS4 primer. The fluorescent-labelled fragments were purified from the unincorporated terminators using the BigDye® XTerminator™ purification protocol. The samples were re-suspended in distilled water and subjected to electrophoresis in an ABI PRISM® 3730XL sequencer (Applied Biosystems). The sequences obtained were manually checked and edited using Finch TV_1_4_0. Query sequences were compared with sequences on the UNITE and NBCI databases to identify the species of ectomycorrhiza; all but one of the sequences had a similarity of over 97 % (Table S1). The sequences were deposited in GenBank with Accession No. KX289956 - KX290005. Morphotypes could not be identified using the DNA analysis and were labelled as unknown.

### Net nitrogen mineralization

To determine net N-mineralization, a 28 day laboratory incubation was carried out using soil collected in 2014. To assess initial and final extractable ammonium (NH_4_^+^), and nitrate (NO_3_^−^), 5 g of fresh soil (2 mm sieved) was extracted with 50 ml 2 M KCl by shaking for 2 h on a reciprocating shaker at 22 rpm. The samples were then allowed to stand for 10 min, and filtered through a Whatman 42 filter paper. NH_4_^+^ and NO_3_^−^ were then determined on a FIA5000 analyzer. Total organic C and total dissolved N in the extracts were determined using a TOC-L SHIMADZU analyzer. For the incubation, another 5 g fresh soil was filled into 100 ml polypropylene tubes, and sealed with Parafilm, which allowed air exchange but retarded moisture loss. The tubes were incubated in ingrowth chamber at 20 °C with 24 h light/dark cycle for 28 days, after which the soils were extracted, and NH_4_^+^ and NO_3_^−^ were determined as described as above. Net N-mineralization was calculated as the change in NH_4_-N plus NO_3_-N during the incubation.

### Mycorrhiza and root tip ectoenzyme analysis

Potential ectoenzyme activities were determined using the high-throughput photometric and fluorimetric 96-well black microplate assays described by Pritsch and Garbaye (2011) and Courty et al. (2005). In 2014, roots tips of two dominant ectomycorrhizal taxa and non-mycorrhizal root tips of each tree species were analysed, and in 2015 all dominant ectomycorrhizal taxa of the tree species and ericoid mycorrhizal fine roots (≤200um) from *Rhododendron ferrugineum* were analysed. Four enzyme activities were measured: β-glucosidase (BG, which hydrolyses cellobiose into glucose), N-acetyl-β-D-glucosaminidase (NAG, which breaks down chitin), acid phosphatase (AP, which releases inorganic phosphate from organic matter), and leucine aminopeptidase (LAP, which breaks down polypeptides). The enzymes activities were expressed as pmol mm^−2^ min^−1^ of total surface area of root tips. The total surface area of the root tips was determined after scanning and image analysis using the PC program WinRhizo 2012b Pro (Regent Instr., Quebec, Canada). For *Rhododendron ferrugineum* the fine roots were checked for the presence of mycorrhizas using a careful staining with 5 % blue ink (Pelikan blue) in 5 % acetic acid. On the scanned images the surface area of the roots less than 0.2 mm in diameter, and shown to contain mycorrhizal structures, were used to calculate the surface area.

The raw enzyme activity values were calculated using following formula:

1 $$ \mathrm{activity}\ \left(\mathrm{in}\ \upmu \mathrm{mol}\right)=\frac{\left({\mathrm{fluorescence}}_{\mathrm{sample}}-{\mathrm{fluorescence}}_{\mathrm{subst}.\mathrm{blank}}\right)\bullet \left(\mathrm{reaction}\ \mathrm{volume}\ \mathrm{in}\ \mathrm{ml}\right)}{\left(\mathrm{extinction}\ \mathrm{coeffient}\right)\bullet 1000} $$

Total enzyme activity was calculated using the following formula:

2 $$ \mathrm{total}\ \mathrm{activity}\left(\mathrm{pmol}\ {\mathrm{mm}}^{-2}{ \min}^{-1}\right)=\frac{\left(\mathrm{activity}\ \left(\upmu \mathrm{mol}\right)\right)\bullet {10}^6}{\left(\mathrm{tip}\ \mathrm{surface}\ \mathrm{area}\ \left({\mathrm{mm}}^2\right)\bullet \left(\mathrm{incubation}\kern0.5em \mathrm{duration}\kern0.5em \left( \min \right)\right)\right)} $$

### Soil enzyme activities

Soil enzyme activity was measured on soils taken in 2014 by following the release of 4-methylumbelliferone (MUF) from the respective substrate (cellobiohydrolase, β-glucosidase, N-acetyl-β-D-glucosaminidase and acid phosphatase). For the protease assay, L-leucine-7-amido-4-methyl coumarin (AMC) was used as substrate, and the released coumarin adduct was measured by fluorescence. Cellobiohydrolase (CBH), β-glucosidase (BG), N-acetyl-β-D-glucosaminidase (NAG), acid phosphatase (AP), leucine aminopeptidase (LAP), phenol oxidase (POX) and peroxidase (PER) activities were assayed in soil homogenates following the protocol described by (German et al. 2011). All activities were determined within 48 h of soil sampling. A homogenate was prepared by dispersing 1 g soil in 100 cm^3^ of 100 mM sodium acetate buffer, which was adjusted with acetic to pH 5.5. Briefly, 50 μl of fluorimetric substrate solution (0.5 mM MUF-cellobioside, 1 mM MUF-N-acetyl-β-D-glucosaminide, 2 mM MUF-phosphate, 1 mM Leucine-amino-methylcoumarin) was combined with 200 μl of soil homogenate in a microplate and incubated for 2 h at 20 °C. The reactions of cellobiohydrolase, β-glucosidase, acetyl-β-D-glucosaminidase and acid phosphatase were stopped by the addition of 10 μl of 1 M NaOH, and the amount of fluorescence was immediately determined in a fluorimeter (Multimode Plate Reader, EnSpire) at 365 nm excitation and 460 nm emission. The assay of each enzyme was replicated four times in each plate, and each plate included a standard curve of the product (MUF, AMC), substrate controls, and homogenate controls. For calibration, methylumbelliferyl (MUF) was used for cellobiohydrolase, β-glucosidase, N-acetyl-glucosaminidase and acid phosphatase activity, whereas AMC was used for calibration of leucine aminopeptidase activity. Enzymatic activity (nmols product released h^−1^ g^−1^ dry soil) was calculated from the MUF and AMC standard curve following (German et al. 2011). Phenoloxidase and peroxidase activities were measured photometrically based on standard methods (Kaiser et al. 2010), using L-3, 4-dihydroxyphenylalanin (L-DOPA, Sigma Aldrich) in microplates. Soil suspension was mixed with a 20 mM L-DOPA solution (1:1). After shaking the samples for 10 min, they were centrifuged and pipetted into microplates. For peroxidase assays, all wells received additionally 10 μl of a 0.3 % H_2_O_2_ solution, including controls. At the beginning and after 20 h, absorption was measured at 450 nm. Enzyme activity was calculated from the increase in absorption over time divided by the molar extinction coefficient.

### Data analysis

Statistical analyses of the data were performed using SPSS 19 program (ANOVA). T-tests were used for detecting differences between vegetation species and elevations, means and standard errors of soil parameters were calculated from each plot. We refer to a *P* value of ≤0.05 as statistically significant, and indicate in some cases a *P* value of ≤0.1 as marginally significant.

Canonical correspondence analysis (CCA) was performed by using Canoco 4.5 software to analyze the ectomycorrhizal community distribution patterns and to test whether ectomycorrhizal community composition was related to the environmental variables (elevation, soil moisture, total C and total N). The plant host was coded as a dummy variable, i.e., 0 and 1. The statistical significance of the environmental variables was evaluated by manual forward selection using a Monte Carlo permutation test with 499 permutations.

## Results

There were significant differences in soil pH, percentage C and N, the C/N ratio and soil temperature between the lower elevation and tree line in *Picea abies* sites (Table 1). At the lower elevation, the soil pH was 0.2 pH units less than at the tree line, and the values of soil C, N, the C/N ratio and soil temperature were significantly higher. At tree line site, significant differences were also found in pH, C, N, C/N ratio, temperature, and moisture of the soils taken from under the tree species and *Rhododendron ferrugineum* plots (Table 1). The soil pH, soil moisture, C, N, C/N ratio and soil temperature did not differ between the *Picea abies* and *Pinus mugo* plots, but were significantly higher under *Rhododendron ferrugineum.*

**Table 1:** Chemical properties of soils taken from under *Picea abies, Pinus mugo* and *Rhododendron ferrugineum* islands at the tree line (1668 to 1791 m) and a *Picea abies* site at a lower elevation (1395 m) at Wasserberg in the central Alps, Austria. Mean ± SE. Data points within a row not followed by the same letter are significantly different (*P* ≤ 0.05) between species or elevation

	*Picea abies*	*Picea abies*	*Pinus mugo*	*Rhododendron ferrugineum*
	Lower elevation	Tree line		
Soil temperature (°C)	11.5 ± 0.1a	9.6 ± 0.2b	9.9 ± 0.2b	11.0 ± 0.3a
pH (H_2_O)	4.4 ± 0.1a	4.6 ± 0.1b	4.6 ± 0.1b	4.8 ± 0.1c
Soil moisture %	54.2 ± 3.2ab	41.4 ± 3.33a	46.2 ± 1.9a	56.6 ± 2.0b
C%	30.5 ± 2.9a	15.5 ± 1.8b	15.7 ± 1.5b	24.3 ± 1.2a
N%	1.46 ± 0.14a	0.91 ± 0.10b	0.87 ± 0.07b	1.20 ± 0.05a
C/N	20.9 ± 0.5a	17.0 ± 0.6b	18.1 ± 0.7b	20.3 ± 0.6a

### Comparison of mycorrhiza community structure between different species

**Fig. 1:**
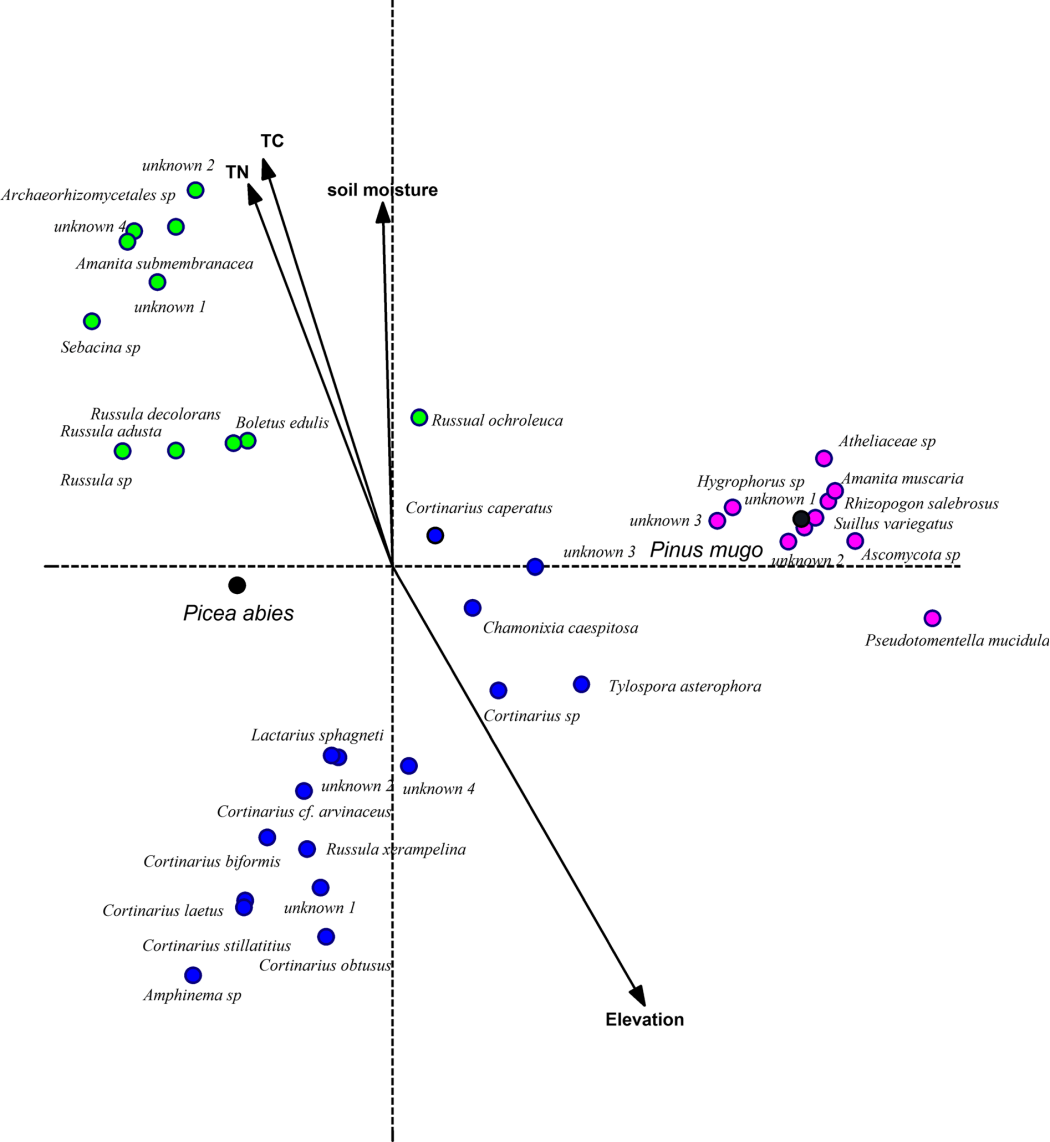
Canonical correspondence analysis diagram of ectomycorrhizal community structure at Wasserberg in the central Alps, Austria. Environment factors used to constrain ordination axes are represented by arrows, dummy variable are represented by *black dots*. Ectomycorrhizal community of *Picea abies* at the lower elevation (1395 m) and the tree line (1668 to 1791 m) are shown by *green and blue closed circles* respectively, Ectomycorrhizal community of *Pinus mugo* is shown by *pink closed circles*. *Russula ochroleuca* occurred at all three sites, and *Continarius caperatus, Russula adusta, Russula decolorans*, *Lactarius sphagneti* were common in *Picea abies* at the tree line and the lower elevation site

In order to separate the mycorrhizal communities, a canonical correspondence analysis (CCA) was carried out (Fig. 1). This showed that ectomycorrhizal communities were separated distinctly by elevational gradient and different host tree species, the first ordination axis (λ_1_ = 0.686, species-environment correlation = 0.925) reflects the host tree species factors, there was strong difference in the ectomycorrhizal communities between the *Picea abies* and *Pinus mugo* at the tree line. At the tree line, in *Picea abies* (Fig. 1) the ectomycorrhizal community was dominated by three *Cortinarius* species. However, on roots of *Pinus mugo* at the tree line the dominant species were *Amanita muscaria* and *Russula orchroleuca* (Fig. 1), together with an unidentified ascomycete.

The second ordination axis of CCA based on ectomycorrhizal (λ_2_ = 0.556, species-environment correlation = 0.869) differentiates species composition between the two sites. Species toward the top of diagram were more abundant at the lower elevation *Picea abies* site which is dominated by *Russula*, conversely, at the bottom of diagram, *Cortinarius* species were abundant at the tree line *Picea abies* site (Fig. 1).

### Soil enzyme activity

**Fig. 2:**
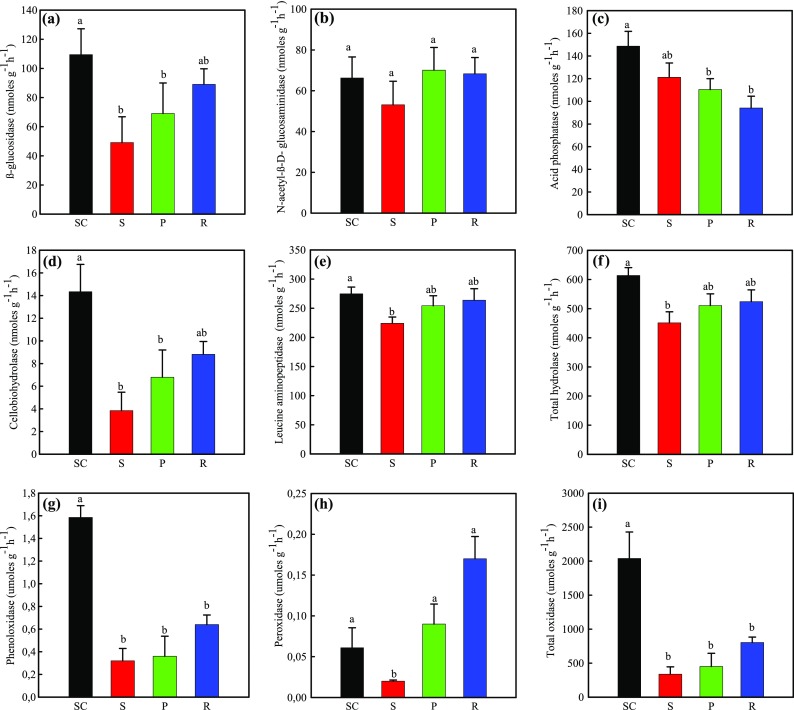
Activity of enzymes in soils taken from under islands of *Picea abies, Pinus mugo* and *Rhododendron ferrugineum* at the tree line (1668 to 1791 m) and a *Picea abies* site at a lower elevation (1395 m) in July 2014. Shown are (**a**) β-glucosidase, (**b**) N-acetyl-ß-D-glucosaminidase, (**c**) Acid phosphatase, (**d**) Cellobiohydrolase, (**e**) Leucine aminopeptidase, (**f**) Total hydrolase, (**g**) Phenol oxidase, (**h**) Peroxidase, (**i**) Total oxidase. Bars show means ± SE. Bars not followed by the same letter are significantly different (*P* ≤ 0.05) between species (**a**, **b**). SC: *Picea abies* at the lower elevation; S: *Picea abies* P: *Pinus mugo* R: *Rhododendron ferrugineum* at tree line site

Enzyme activities in the soils varied widely between elevation and the dominating vegetation at the tree line (Fig. 2). For soils taken from under *Picea abies,* significantly higher activities of β-glucosidase (Fig. 2a), peroxidase (Fig. 2h), cellobiohydrolase (Fig. 2d), leucine amino peptidase (Fig. 2e), phenol oxidase (Fig. 2g), total hydrolase (Fig. 2f) and total oxidase (Fig. 2I) were found at the lower elevation site than the tree line site. The activities of N-acetyl-glucosaminidase (Fig. 2b) and acid phosphatase (Fig. 2e) in soils did not differ under *Picea abies* between the different elevations. At the tree line site, the enzyme activity of peroxidase (Fig. 2h) was significantly higher in soil under *Rhododendron ferrugineum* and that from under *Picea abies*. The enzyme activities of β-glucosidase, cellobiohydrolase, leucine aminopeptidase, phenol oxidase, total hydrolase and total oxidase consistently increased in the order *Picea abies* < *Pinus mugo* < *Rhododendron ferrugineum*, the acid phosphatase activity in soils exhibited an opposite tendency to the other soil enzyme activities, which decreased in order *Picea abies* < *Pinus mugo* < *Rhododendron ferrugineum* (Fig. 2g). However, no significant differences were found between the different species.

### Mycorrhizal root enzyme activity

**Fig. 3:**
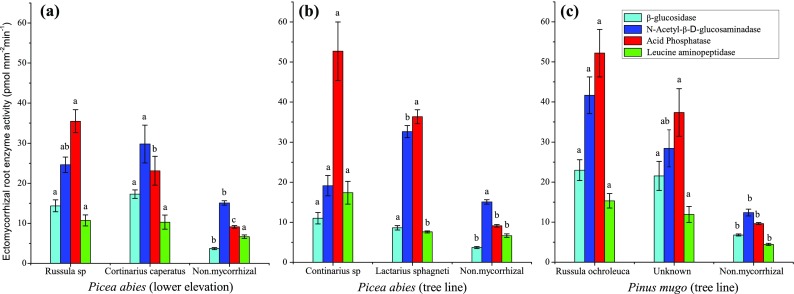
Enzyme activities of ectomycorrhizal roots tips and non-mycorrhizal root tips of trees at two elevations. (**a**) *Picea abies* (SC) at the lower elevation (1395 m), (**b**) *Picea abies* (S) at tree line, (**c**) *Pinus mugo* (P) at tree line (1668 to 1791 m). The roots were collected at Wasserberg in the central Alps, Austria in July 2014. Bars show means ± SE. Within a tree species, for each enzyme activity, bars not followed by the same letters are significantly different (*P* ≤ 0.05) between ectomycorrhizal species or non-mycorrhizal roots tips (**a**, **b**)

With exception of N-acetyl-glucosaminadase from *Picea abies* at the tree line site and leucine aminopeptidase from *Picea abies* at the lower elevation, the enzyme activities of the non-mycorrhizal root tips were significantly lower than at least one, and mostly both, of the two ectomycorrhizal fungal tested (Fig. 3). In 2014, for each tree species or elevation, two different taxa of ectomycorrhizal fungi were investigated. At the lower elevation *Picea abies* site, between the two taxa of ectomycorrhizal fungi investigated, the activity of only one enzyme, acid phosphatase, was significantly different between *Continarius caperatus* and *Russula sp.* (Fig. 3a). At the tree line, in *Picea abies*, the enzyme activity of leucine aminopeptidase was significantly higher in *Continarius sp.* root tips than in *Lactarius sphagneti* root tips (Fig. 3b). Whereas the activity of N-acetyl-ß-D-glucosaminidase was lower *Continarius sp.* root tips than in *Lactarius sphagneti* root tips. In *Pinus mugo* (Fig. 3c), no significant difference in the activities of the four enzymes investigated were found between the two ectomycorrhizal taxa *Russula ochroleuca* and an unidentified species (unknown 1).

**Fig. 4:**
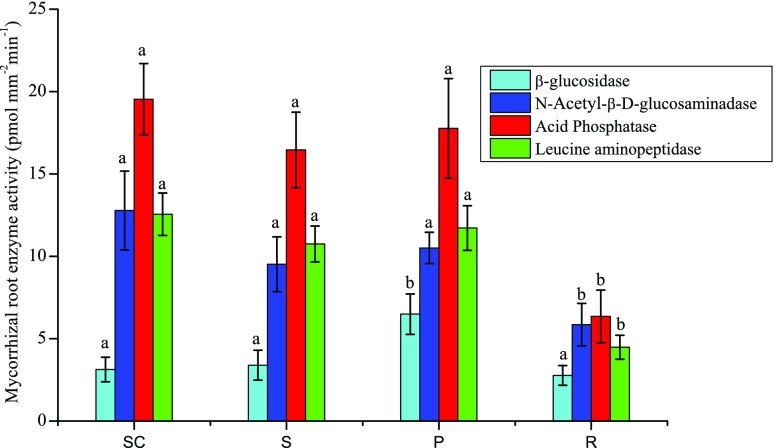
Mean root enzyme activities of all ectomycorrhizal taxa of (SC) *Picea abies* at the lower elevation (1395 m), (S) *Picea abies* at the tree line, (P) *Pinus mugo* at the tree line, and (R) from hair roots of *Rhododendron ferrugineum* at the tree line (1668 to 1791 m). The roots were collected at Wasserberg in the central Alps, Austria on 12th June 2015. Bars show means ± SE. Within an enzyme activity, bars not followed by the same letters are significantly different between species or elevation (*P* ≤ 0.05)

To better estimate the enzyme activity for the complete spectrum of ectomycorrhizas found on the roots, in 2015 the enzyme activities of all dominant ectomycorrhizal taxa were determined for *Picea abies* and *Pinus mugo*. In total, twelve ectomycorrhizal taxa from *Picea abies* at tree line and lower the elevation were determined and 7 ectomycorrhizal taxa from *Pinus mugo*. In addition, the enzyme activities were determined on four samples of fine roots from *Rhododendron ferrugineum* (Fig. S2). Between the years the absolute levels of activity of each of the enzymes were similar, but between taxa the levels of activity varied several fold for all enzymes (Fig. S2). Averaged across all taxa of ectomycorrhizal species no significant differences were shown in any of the enzymes between tree species or elevation except that β-glucosidase activity was significant higher in *Pinus mugo* (Fig. 4). On the fine roots of *Rhododendron ferrugineum* the activities of N-acetyl-ß-D-glucosaminidase, acid phosphatase, and leucine aminopeptidase were significantly lower compared to the ectomycorrhizal taxa of the tree roots. The activity of β-glucosidase on *Rhododendron ferrugineum* was similar to activity determined on the ectomycorrhizal taxa of *Picea abies* (Fig. 4).

### Total N, total C, N-mineralization, decomposition rate

The extractable levels of total dissolved N, dissolved organic N (DON), NH_4_^+^, NO_3_^−^ and dissolved organic C (DOC) were determined in the soils directly after sampling. At all sites, dissolved N was mainly in the form of DON, and a smaller amounts were present in the form of NH_4_^+^, only very low levels of NO_3_^−^ were determined. At tree line site, total dissolved N, DON, and DOC were significant higher in soils from *Picea abies* than from soils taken from under *Pinus mugo* and *Rhododendron ferrugineum* (Table 2). For *Picea abies,* no significant differences in any parameters were found between the different elevations.

**Table 2:** The concentration of dissolved total N, organic and inorganic N fractions, and DOC, as well as the DOC to DON ratio in soils taken from under islands of *Picea abies*, *Pinus mugo*, *Rhododendron ferrugineum* at the tree line (1668 to 1791 m) and a *Picea abies* site at a lower elevation (1395 m) at Wasserberg in the central Alps, Austria. Also shown are rates of net N-mineralization (25 ° C for 28 days) of the soils taken from under the patches, and the mass loss of decomposition bags filled with two types of material green tea or red tea placed at 5cm depth in the soil under the islands for 120 days. Mean ± SE. Within a parameter, means not followed by the same letters are significantly different (*P* ≤ 0.05) between species or elevation

	*Picea abies*	*Picea abies*	*Pinus mugo*	*Rhododendron ferrugineum*
	Lower elevation	Tree line		
Total dissolved N (mg kg^−1^)	91.2 ± 7.7a	99.8 ± 10.0a	76.5 ± 6.1b	68.1 ± 2.5b
DON (mg kg^−1^)	83.6 ± 5.9 a	90.8 ± 9.2 a	61.5 ± 7.7 b	62.1 ± 2.2 b
NH_4_^+^ (mg kg^−1^)	6.9 ± 1.3a	6.0 ± 1.5a	14.7 ± 4.2a	5.5 ± 1.2a
NO_3_^−^ (mg kg^−1^)	0.49 ± 0.12a	2.98 ± 1.19a	0.34 ± 0.08a	0.47 ± 0.01a
DOC (mg kg^−1^)	612 ± 50a	684 ± 120a	478 ± 48b	467 ± 10b
DOC /DON	13.5 ± 3.5a	9.2 ± 0.6a	7.8 ± 0.5a	8.2 ± 0.8a
N-Mineralization mg kg^−1^ day^−1^	3.7 ± 0.7a	2.5 ± 0.4a	1.5 ± 0.2ab	1.0 ± 0.4b
N-Mineralization mg N kg^−1^ SOC day^−1^	12.4 ± 2.6a	17.9 ± 3.6a	10.1 ± 1.6a	2.4 ± 1.2b
Percentage mass loss (green tea)	64 ± 1a	53 ± 2b	57 ± 2b	56 ± 1b
Percentage mass loss (red tea)	29 ± 1a	25 ± 1b	28 ± 2ab	25 ± 1b

Nitrogen mineralization rate did not differ in *Picea abies* between the different elevations, but at the tree line, the rate of N-mineralization was significantly lower in soil from under *Rhododendron ferrugineum* than in soil from under *Picea abies.* Nitrogen mineralization rate was less than half in soil from under *Rhododendron ferrugineum* than that from under *Picea abies.*

Decomposition rate measured as either mass loss from green tea or more recalcitrant red tea was higher at the lower elevation *Picea abies* site compared to the tree line site. Between the species at the tree line no significant differences in decomposition rate were observed (Table 2).

## Discussion

### Ectomycorrhizal community structure

In *Pinus mugo* at the tree line, the ectomycorrhizal community was dominated by *Amanita muscaria* and *Russula orchroleuca*, whereas the ectomycorrhizal community associated with *Picea abies* site was dominated by *Continarius sp*. As these tree species co-exist and are subject to the same environmental conditions, this difference in ectomycorrhizal community suggests a clear influence of host (Massicotte et al. 1994), especially as the properties of soil under the two tree species were identical. At lower elevation *Picea abies* site, the ectomycorrhizal community was dominated by taxa from the genus *Russula*. The difference in community structure between the higher and lower elevation sites of *Picea abies* could be due to a number of factors including tree age, but also some soil properties. The *Picea abies* trees at the lower elevation were determined to be between 90 and 120 years old, but at the tree line only 30 years old. The importance of host plant age on ectomycorrhizal communities was shown by Mason et al. (1982, 1983), who developed the concept of early and late successional stage fungi. Although it is clear that tree age can have impacts on ectomycorrhizal fungal communities, the mechanisms behind this are unclear (Johnson et al. 2005). There were also differences in pH, soil moisture, C and N of the soils from the higher and lower elevation sites of *Picea abies*, all of which can influence ectomycorrhizal community structure (Aggangan et al. 1996; Kainulainen et al. 1996; Lodge 1989; Parrent et al. 2006). The ectomycorrhizal community of the higher elevation site was dominated by medium distance exploration type (*Continarius sp.*), whereas the ectomycorrhizal community of the lower elevation site was dominated by contact exploration types (*Russula*). Agerer (2001, 2007) has suggested that harsher sites (dry, low nutrient level) tend to have long or medium distance exploration types and wetter sites have more often contact exploration types. However, Peay et al. (2011) could show that long distance exploration types are more common in areas of low tree root density and at the edges of tree islands.

### Root ectoenzymes

Comparison of ectoenzyme activity of the different ectomycorrhizal taxa to non-mycorrhizal root tips shows that the ectomycorrhizal taxa generally have a higher activity for all the enzymes estimated, but also that non-mycorrhizal root tips have considerable activity. However, although there were strong differences in the ectomycorrhizal community structure of the *Picea abies* and *Pinus mugo* roots, and differences in enzyme activity of the individual taxa of the ectomycorrhizas, the mean activity across all ectomycorrhizal taxa is similar for all the 4 enzymes with the exception of β-glucosidase in *Pinus mugo.* Thus the contribution of the different ectomycorrhizal communities to the soil enzyme pool is similar irrespective of the ectomycorrhizal community structure.

The majority of measurements of enzyme activity of ericoid mycorrhizas have been carried out on in vitro cultures (Kerley and Read 1998; Leake and Read 1990), and we could find no reported measurements of root surface enzyme activity of ericoid mycorrhizal roots. Surprisingly, the roots surface ectoenzyme activity of hair roots of *Rhododendron ferrugineum* was with the exception of β-glucosidase lower than that of the mean activity of the tree species. Thus this is in contrast to the idea (Read et al. 2004) that ericoid fungi are strongly involved in mobilization of N from recalcitrant organic matter in soils through production of enzymes such as N-acetyl-ß-D-glucosaminidase and leucine aminopeptidase. A potential reason for the lower ectoenzyme activity in the hair roots of *Rhododendron ferrugineum* could be the lower amount of surface fungal tissue in the ericoid compared to the ectomycorrhizal roots. Although the hair roots of *Rhododendron ferrugineum* were clearly infected with ericoid fungi, the enclosed ericoid structures will have a lower exposed fungal surface area than the hyphal mantel of the ectomycorrhizas. However, in soils, fine roots of ericoid plants have a high proliferation (Wurzburger and Hendrick 2007) and can form dense root mats (Read et al. 2004) whereas the ectomycorrhizal roots are more dispersed. Thus in soils the higher root surface area may compensate for the lower surface area enzyme activity in *Rhododendron ferrugineum*.

### Soil enzyme activity, decomposition and N- mineralization

Between the *Picea abies* sites at the different elevations in soils there were significant differences in the activity of phenol oxidase, peroxidase, β-glucosidase and cellobiohydrolase, all the enzymes involved in degradation of organic matter. The higher activity of these enzymes in the soil at the lower elevation site is reflected in the highest mass loss of both the easily decomposable green tea bags and the more recalcitrant red tea bags. The several fold higher activity of oxidases in the soil of the lower elevation *Picea abies* site could be due to more developed forest floor and higher activity of saprotrophic fungi (Baldrian 2009), this is consistent with the greater mass loss. The differences found in mass loss and potential enzyme activity may also be due to differences in soil temperature. Soil temperature was on average 1 °C higher at the lower elevation site. Even small changes in soil temperature affect the activity of enzymes in forest soils (Baldrian et al. 2013), in particular cellobiohydrolase was very temperature sensitive in the range 5 to 15 °C. In other studies soil temperature was the strongest factor driving low in situ enzyme activities in the Arctic, and warming increased soil enzyme activities in winter (Ajwa et al. 1999; Wallenstein et al. 2009). However, the higher amounts of C and higher C/N ratio of soils at the lower elevation *Picea abies* site suggest that the input of organic matter exceeds the rates of decomposition. Lower C/N ratios in soil, as found in the higher elevation *Picea abies* plots, are often associated with more processed soil organic matter (Dick 1983; Gregorich et al. 1994).

In the soil of the different vegetation islands at the tree line, the activities of phenol oxidase, peroxidase, β-glucosidase and cellobiohydrolase consistently increased in the order *Picea abies*<*Pinus mugo*<*Rhododendron ferrugineum*, however, no significant difference were found for the individual enzymes. The lack of significant differences in soil enzyme activity between the different vegetation types is consistent with that for both of the types of decomposition bags used a similar mass loss was also found between the vegetation types. For the enzymes involved in mobilization of N, N-acetyl-ß-D-glucosaminidase and leucine aminopeptidase, the activity in the soil under *Picea abies*, *Pinus mugo* or *Rhododendron ferrugineum* was similar. As the root surface activity of these enzymes was significantly lower in *Rhododendron ferrugineum* compared to *Picea abies* and *Pinus mugo*, this again suggests that high root biomass may compensate for the lower root surface activity in *Rhododendron ferrugineum*. The rate of net N-mineralization was lower in soil from under *Rhododendron ferrugineum* than in soil from under *Pinus mugo* and *Picea abies*. The lower rate of N-mineralization corresponds to significantly lower levels of DON and DOC under *Rhododendron ferrugineum* compared to *Picea abies*. As the enzyme activities of N-acetyl-ß-D-glucosaminidase and leucine aminopeptidase were similar between the 3 vegetation types, the lower N-mineralization and lower DON and DOC suggest that an aspect litter quality limits decomposition under *Rhododendron ferrugineum.* A number of *Rhododendron* species have been shown to have low levels of N in leaves and high C/N ratios in litter (Cornelissen et al. 2001; Wurzburger and Hendrick 2007). The presence of ericaceous plants like *Rhododendron maximum* leads to accumulation of organic matter (Clemmensen et al. 2015; Wurzburger and Hendrick 2007), due to high and recalcitrant leaf and root litter inputs (Wurzburger and Hendrick 2007). In addition, ericoid plants produce tannin and polyphenol-rich leaf litter and root litter that inhibit N-mineralization (Boettcher and Kalisz 1990) and rates of nitrification (DeLuca et al. 2002). This is in accordance with the low rate of N-mineralization shown in the soil form under *Rhododendron ferrugineum*. Tannic acid extract from *Rhododendron maximum* litter cause protein to precipitate, although the amount of precipitation was not greater than that caused by extracts from a number of tree species (Wurzburger and Hendrick 2007). Bending and Read (1996a, 1996b, 1997) examined the abilities of the ericoid fungi *Hymenoscyphus ericae* and an *Oidiodendron sp.* to gain access to organic N after it had been co-precipitated as protein with tannic acid, and showed that sufficient access to this N was gained to support growth of the fungus. If this is a specific ability of ericoid mycorrhizal fungi to release N, it is not reflected in net rates of N-mineralization. However, the recalicitrance of the *Rhododendron ferrugineum* litter is reflected in the higher C/N ratio of the soil which again suggests that the soil organic matter is less processed (Dick 1983; Gregorich et al. 1994) with inputs rates of litter exceeding decomposition. Baldrian (2009) suggested that litter from species that contain more recalcitrant material limits the C supply to the microbial community and results in a higher potential activity of enzymes involved in the degradation of cellulose and proteins, acquisition of phosphate, and oxidation of phenols, which is a possible explanation for the higher values of activity of phenol oxidase, peroxidase, β-glucosidase and cellobiohydrolase activity in soil under *Rhododendron ferrugineum*. Recently, Clemmensen et al. (2015) suggested that ericoid mycorrhizal fungi may lock up more C and nitrogen than they release from the long term soil organic matter pool, primarily as a result of impaired decomposition of their necromass. Phuyal et al. (2008) reported that phosphatase activity increased as N availability increased and decreased with addition of P. These authors also suggested a positive relationship between phosphatase activity and tissue N concentration (Phuyal et al. 2008). In the soil under *Rhododendron ferrugineum* the lowest phosphatase activity of all the vegetation types was found corresponding to the lowest total dissolved N.

## Conclusions

The results of this study show that despite the difference in ectomycorrhizal community structure and even across changes in mycorrhizal type, from ectomycorrhizas to ericoid mycorrhizas, for ecosystem function measured in terms of decomposition, N-mineralization and soil enzyme activities, the similarities outweigh the differences. This is particularly obvious if the ectomycorrhizal communities of *Pinus mugo* and *Picea abies* at the tree line are compared. In these vegetation islands very similar values for the ecosystem function parameters were determined although the ectomycorrhizal communities differed. Jones et al. (2010) came to similar conclusion in *Pseudotsuga menziesii* forests after disturbance through wild-fire or clear cutting. Although after disturbance different ectomycorrhizal communities were found, the activity of rhizoplane enzymes was similar. However as the *Picea abies, Pinus mugo* and *Rhododendron ferrugineum* co-exist at the tree line the environment conditions and parent geology are similar, with the exception of differences in the quality of litter inputs, thus there is no *a priory* reason to assume that ecosystem function should differ. Rather our results suggest that different combinations of species and types of mycorrhizal fungi, albeit with a potentially high functional plasticity, can have a similar ecosystem function in soils.

## Electronic supplementary material

Fig. S1 (PDF 417 kb)

Fig. S2 (PDF 236 kb)

Table S1 (PDF 21 kb)
