# Supplementary material for: Mycorrhizas and soil ecosystem function of co-existing woody vegetation islands at the alpine tree line
Source: Plant Soil. 2016 Sep 12;411(1):467–81. doi: 10.1007/s11104-016-3047-2 (PMC5288427; doi:10.1007/s11104-016-3047-2)

Fig. S1 Images of the tree line site (1668 to 1791 m) at Wasserberg in the central Alps, Austria. **Top left**, a typical soil profile. **Top right**, a view across the site. **Bottom right**, a bush of *Rhododendron ferrugineum*. **Bottom left**, in the foreground *Rhododendron ferrugineum*, in the middle of the picture *Pinus mugo*, and at the back of the picture *Picea abies*.

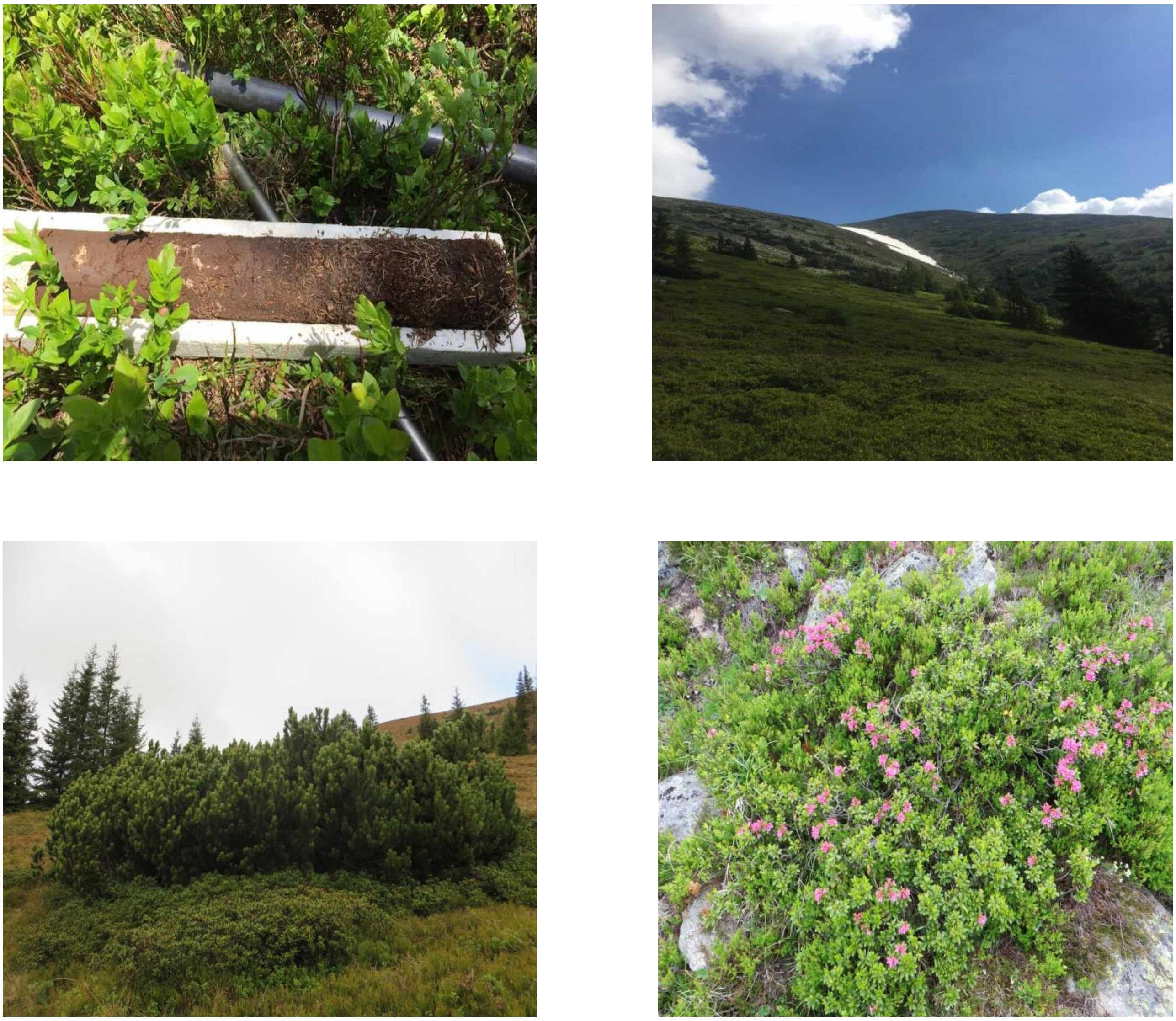

Supplement: Supplementary file 1 — (PDF 417 kb) [file 11104_2016_3047_MOESM1_ESM.pdf]
