# Supplementary material for: Mycorrhizas and soil ecosystem function of co-existing woody vegetation islands at the alpine tree line
Source: Plant Soil. 2016 Sep 12;411(1):467–81. doi: 10.1007/s11104-016-3047-2 (PMC5288427; doi:10.1007/s11104-016-3047-2)

Fig. S2 Roots enzyme activities of all domainated mycorrizal taxa on roots of (a) *Picea abies* (SC) at the lower elevation (1395 m), (c) *Picea abies* (S) at the tree line (1668 to 1791 m), (d) *Pinus mugo* (P) at the tree line (1668 to 1791 m), and (b) from fine roots of *Rhododendron ferrugineum* (R) at the tree line (1668 to 1791 m). The roots were collected at Wasserberg in the central Alps, Austria on June 12th 2015. Bars show means ± SE. SC1 *Thelephora terrestris*, SC2 *Boletus edulis*, SC3 *Tylospora asterophora*, SC4 *Russula decolorans*, SC5 *Inocybe palaeotropica*, SC6 Unknown 1, SC7 *Russula sp*, SC8 *Tylospora asterophora*, SC9 *Russula adusta*, SC10 *Cortinarius sp*, SC11 *Russula ochroleuca*, SC12 Unknown 2, SC13 Unknown 3; S1 Unknown 1, S2 *Tylospora sp*, S3 *Cortinarius sp*, S4 *Continarius laetus*, S5 Unknown 2, S6 Unknown 3, S7 *Amphinema sp*, S8 *Cortinarius obtusus*, S9 *Pseudotomentella mucidula*, S10 Unknown 4, S11 *Amphinema byssoides*, S12 *Chamonixia caespitosa*, P1 *Atheliaceae*, P2 *Russulaceae*, P3 *Boletus edulis*, P4 *Ascomycota sp*, P5 *Suillus variegatus*, P6 *Russula sp*, P7 *Atheliaceae*, P8 *Amanita muscaria*.

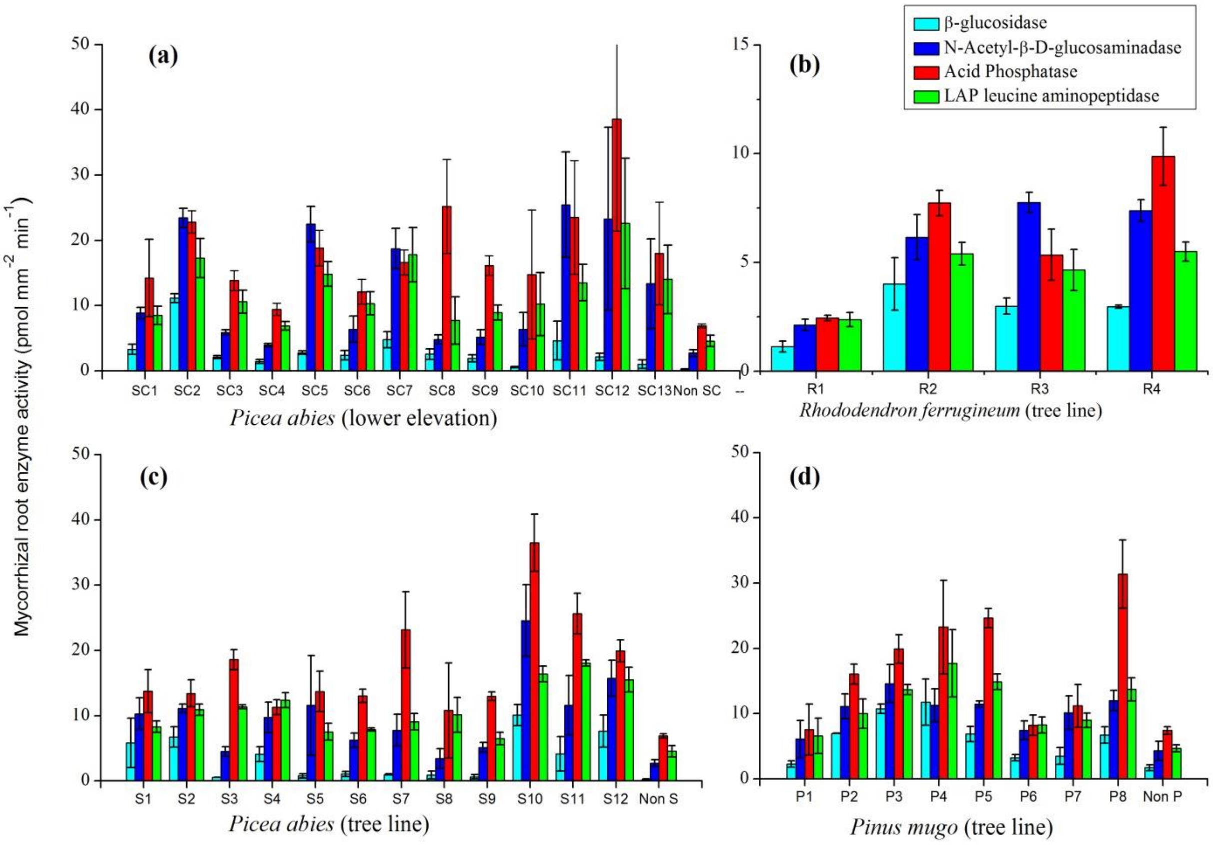

Supplement: Supplementary file 2 — (PDF 236 kb) [file 11104_2016_3047_MOESM2_ESM.pdf]
