# Supplementary material for: Mycorrhizas and soil ecosystem function of co-existing woody vegetation islands at the alpine tree line
Source: Plant Soil. 2016 Sep 12;411(1):467–81. doi: 10.1007/s11104-016-3047-2 (PMC5288427; doi:10.1007/s11104-016-3047-2)
Supplement: Supplementary file 3 — (PDF 21 kb) [file 11104_2016_3047_MOESM3_ESM.pdf]

Table S1. Successfully sequenced EM fungi using the primer ITS4

Accession	Closest Genbank/Unite Match	Accession	Coverage (%)	Similarity (%)
KX289956	*Russula ochroleuca*	KT933996	99.6%	98%
KX289957	*Russula ochroleuca*	UDB016009	99.8%	99%
KX289958	*Russula ochroleuca*	KT933996	99.4%	97%
KX289959	*Russula decolorans*	UDB011326	99.8%	97%
KX289960	*Russula decolorans*	UDB011326	99.8%	98%
KX289961	*Russula decolorans*	KT933992	99.7%	99%
KX289962	*Russula adusta*	UDB023484	99.8%	98%
KX289963	*Russula adusta*	UDB023484	98.4%	98%
KX289964	*Russula sp*	JF519262.1	97%	98%
KX289965	*Russula xerampelina*	UDB015068	99.6%	96%
KX289966	*Chamonixia caespitosa*	KT001259	99.8%	99%
KX289967	*Cortinarius caperatus*	DQ367911	99.9%	99%
KX289968	*Cortinarius laetus*	UDB001046	99.8%	99%
KX289969	*Cortinarius biformis*	UDB002252	99.7%	99%
KX289970	*Suillus variegatus*	UDB015800	98.9%	97%
KX289971	*Cortinarius cf. arvinaceus*	HQ604690.1	98.9%	98%
KX289972	*Amanita muscaria*	AB096048	99.8%	99%
KX289973	*Tylospora asterophora*	KF618042.1	99%	95%
KX289974	*Lactarius sphagneti*	KT165247	99.4%	98%
KX289975	*Amanita submembranacea*	UDB002350	99.8%	99%
KX289976	*Atheliaceae sp*	EU645645	99%	98%
KX289977	*Cortinarius stillatitius*	UDB020290	100%	100%
KX289978	*Amphinema sp*	KF305820.1	99%	96%
KX289979	*Ascomycota sp*	JQ711841.1	99%	98%
KX289980	*Archaeorhizomycetales sp*	JN032483	99.8%	99%
KX289981	*Hygrophorus sp*	KJ720200	99.7%	99%
KX289982	*Cortinarius sp*	JX029933.1	99%	99%
KX289983	*Cortinarius sp*	AY641464	99%	98%
KX289984	*Suillus variegatus*	AM084696	99.5%	96%
KX289985	*Pseudotomentella mucidula*	KP783454	99.6%	99%
KX289986	*Sebacina sp*	HM146865	99.6%	97%
KX289987	*Rhizopogon salebrosus*	AF377159.1	99.9%	99%
KX289988	*Cortinarius obtusus*	UDB000127	99.7%	99%
KX289989	*Cortinarius caperatus*	KJ421125	100%	99%
KX289990	*Amphinema sp*	KR019836	99.8%	97%
KX289991	*Cortinarius sp*	DQ481834	99.8%	98%
KX289992	*Russula ochroleuca*	HM189930	99.6%	99%
KX289993	*Thelephora terrestris*	JQ712012	99%	98%
KX289994	*Boletus edulis*	UDB015697	99.9%	99%
KX289995	*Tylospora asterophora*	UDB016384	99.4%	99%
KX289996	*Inocybe palaeotropica*	HQ604523	99.8%	96%
KX289997	*Tylospora asterophora*	UDB008244	100%	99%
KX289998	*Amphinema byssoides*	JN943921	99.3%	96%
KX289999	*Cortinarius sp*	JX029943	99.7%	99%
KX290000	*Amphinema sp*	UDB008312	99.9%	99%
KX290001	*Cortinarius obtusus*	UDB000127	100%	99%
KX290002	*Amphinema byssoides*	JX907809	99.7%	97%
KX290003	*Russulaceae sp*	EF521209	99.9%	99%
KX290004	*Boletus edulis*	UDB015697	99.9%	99%
KX290005	*Atheliaceae sp*	FJ152541	99.8%	99%

The accession numbers and similarity indices refer to the nucleic acid sequences from the ITS region of the rDNA, compared with the UNITE (http://unite.ut.ee) and NCBI GenBank (http://www.ncbi.nih.gov/BLAST) databases using the BlastN algorithm.
